# A Core-Shell MWCNT-Pt Nanowire Electron Source with Anomalously Long-Term Stable Field Emission

**DOI:** 10.3390/nano13030532

**Published:** 2023-01-28

**Authors:** Wenqi Zhang, Peidong Chao, Donglei Chen, Zhan Yang, Lixin Dong

**Affiliations:** 1 Jiangsu Provincial Key Laboratory of Advanced Robotics, School of Mechanical and Electric Engineering, Soochow University; Suzhou 215000, China; 2 Department of Biomedical Engineering, City University of Hong Kong, Hong Kong 999077, China

**Keywords:** field emission, hybrid nanowire, electron source, focused electron-beam-induced deposition, nanorobotic manipulation

## Abstract

A hybrid core-shell structured nanowire is proposed for a long-term stable electron source based on an isolated platinum/multi-walled carbon nanotube (Pt/MWCNT). This hybrid nanowire is prepared by growing a Pt shell on a metallic MWCNT through a field-emission-induced deposition (FEID) method. An in situ field emission (FE) platform was constructed inside a scanning electron microscope (SEM) equipped with two nanorobotic manipulators (NRMs) for the preparation and testing of the hybrid nanowire. An in situ fatigue test was conducted with high current intensity (500 nA) to show the influence of the Pt shell. Compared with the pristine bare MWCNT, our hybrid-nanowire-based electron source has a lifetime of hundreds of times longer and can work continuously for up to 48 h under relatively high pressure ($3.6\times 10^{-3}$ Pa) without having an apparent change in its structure or emission currents, demonstrating good stability and tolerance to poor working conditions. The anomalous long-term stability is attributed mainly to the shielding of oxygen by Pt from the carbon shells and less heating due to the work function lowering by Pt.

## 1. Introduction

Electron source plays an important role in the development of advanced instruments and functional devices, such as electron microscopes [1], microwave amplifiers [2], field emission display (FED) [3,4], etc. Since de Heer demonstrated the excellent field emission (FE) properties of carbon nanotubes (CNTs) in 1995 [5], CNT emitters have received wide attention for their large aspect ratio, nanoscale tip, chemical stability, and excellent thermal conductivity [6], and are expected to be the ideal material for the next generation of electron sources [7,8,9,10,11]. The CNT-based electron source shows excellent properties, especially in high brightness [1], narrow energy distribution [12,13], and stable emission current [14]. 

However, long-term stability, among some other problems, has restricted pristine CNTs as electron sources from practical use [15]. This includes degradation due to dangling bonds [16]; defects; unraveling on the tip caps; gas adsorption [17,18]-induced structural damages; overheating from the poor contact between the CNT and the electrodes, which is considered one of the main reasons for the failure of the CNT-based electron source [19]; the “knock-on” effect induced by the high-power electron beam [20], the piece-by-piece pilling process owing to strong electrostatic force [21], and so on.

To achieve better performance and improve the long-term stability of the CNT- based electron sources, various methods have been developed. For example, boron and phosphorus compounds [22] have been used to modify the surface of CNTs, and various metal films (Ti, Au, Pd, Fe, Al, W, and Pb) are used to coat CNTs to improve their thermal stability [23,24]. However, most research about CNT-based FE has focused on an array or a bulk form with an expectation to use arrayed electron sources to solve the problems of the stability and current intensity of a single CNT. There is an urgent need to realize the long lifetime and structural stability of an isolated CNT.

In this paper, we have grown a Pt shell on the tip of a bare MWCNT through a field-emission-induced deposition (FEID) method [25,26] and achieved excellent stability. A high-density emission current (500 nA) can be extracted with a working voltage of less than 200 V under a vacuum of $3.6\times 10^{-3}$ Pa. Next, we will describe our work from the perspectives of test platform construction, nanowire preparation, and fatigue testing, and then theoretically analyze the effect of the grown Pt shell on the FE enhancement of the hybrid nanowire.

## 2. Materials and Methods

**Figure 1:**
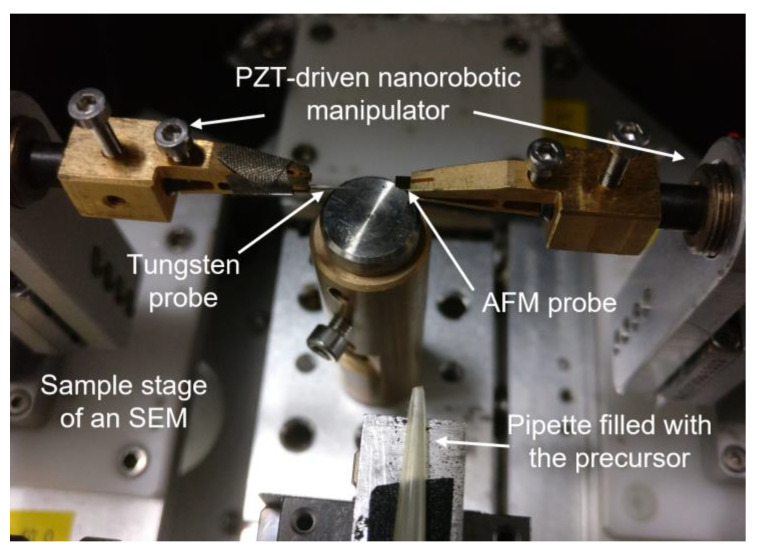
A schematic diagram of the in situ field emission platform we built inside an SEM, with two PZT-driven NRMs in it.

To prepare and test the platinum/multi-walled carbon nanotube (Pt/MWCNT) hybrid-nanowire-based electron source, we built an in situ field emission platform inside a scanning electron microscope (SEM, Merlin compact, Carl Zeiss, Oberkochen, Germany). In this platform, two nanorobotic manipulators (NRMs) [27,28] are installed opposite each other on the sample stage of the SEM. Each manipulator has four lead-zirconate-titanate (PZT) driven actuators, bringing one degree of freedom (DOF) for each and eight DOFs for two manipulators in total. A remote programmable source meter (Model 6430, Keithley, Cleveland, OH, USA) is introduced into the SEM chamber for bias application and precision measurement. An image of the in situ field emission platform is shown in Figure 1. 

**Figure 2:**
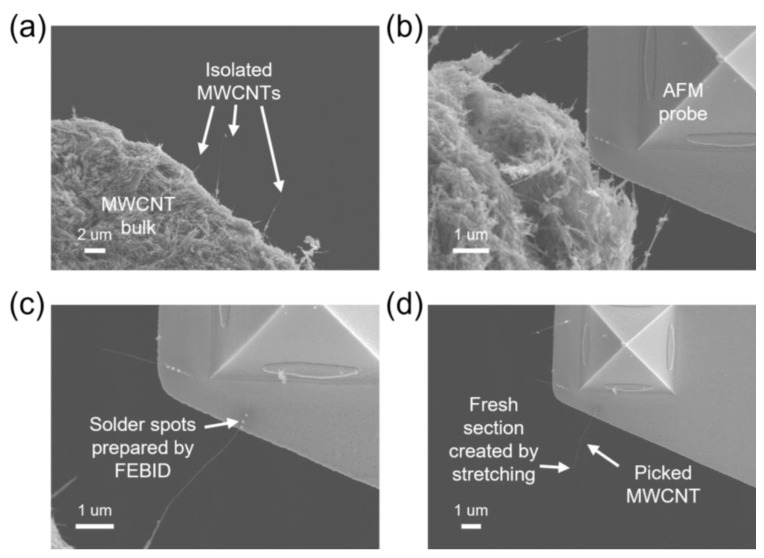
The picking-up process of an isolated MWCNT from the MWCNT bulk with our NRM. (**a**) SEM image of the MWCNT bulk with isolated MWCNT sticking out of the bulk surface; (**b**) approaching process of an AFM probe carried by the NR manipulator; (**c**) welding operation to mount the MWCNT to probe’s surface, where solder spots prepared by the FEBID method can be seen; (**d**) the picked MWCNT was stretched to breaking, generating a fresh section.

The hybrid-nanowire-based electron source is prepared from a free-standing metallic multi-walled CNT (MWCNT). The MWCNTs used in our study were prepared by the arc-discharge method [29] and usually grown on the base in the form of a bundle. Here, we dipped in the MWCNTs with a conductive tape to form an MWCNT bulk, then the bulk was mounted on an NRM. The other NRM was employed to carry an atomic force microscope (AFM) probe. Figure 2 depicts the picking-up process of an MWCNT. At high magnification (greater than 3500×), we can find many isolated MWCNTs on the surface of the bulk, as shown in Figure 2a. Firstly, an MWCNT with a good shape (long and straight) was chosen as the target and moved to the view center of the SEM. The manipulator with an AFM probe was controlled to approach the target MWCNT, as shown in Figure 2b. When the image of the AFM probe was overlaid on the image of the MWCNT, we gradually lifted the AFM probe until it came into contact with the WMCNT. The contact can be judged by the start of the adjoint movement of the MWCNT. A focused-electron-beam-induced deposition (FEBID) method [30] was conducted by the electron beam of the SEM to execute the welding operation for a more reliable mechanical and electrical connection [31]. The precursor used here is W(CO)_6_. Several solder spots of tungsten (W) were deposited on the connection between the MWCNT and the probe’s surface and can be found in Figure 2c. Then, the AFM probe was moved away from the MWCNT bulk, and the MWCNT was stretched and eventually broken, generating a fresh section, as shown in Figure 2d. 

**Figure 3:**
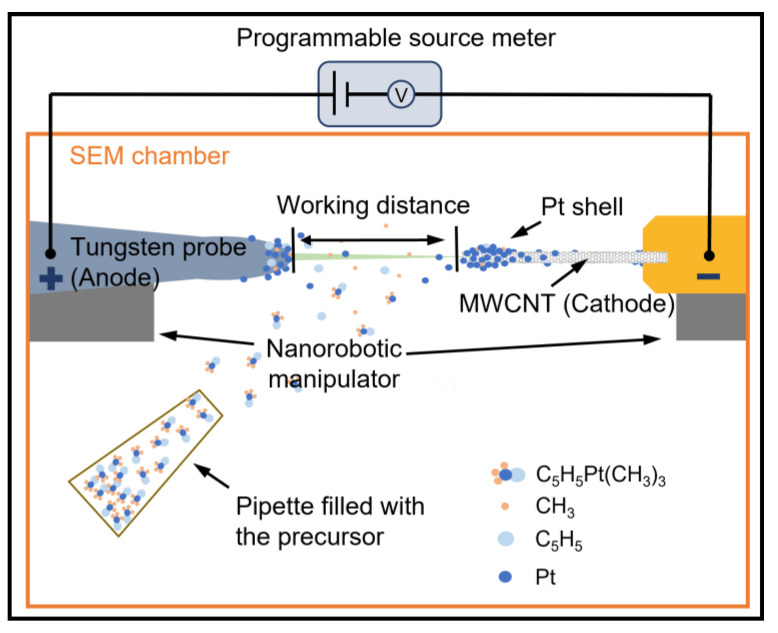
Diagram of the setup for the FE test and in situ growth of the Pt shell inside an SEM.

Next, we replaced the conductive tape with a W probe for an FE test on the pristine MWCNT. Figure 3 shows a diagram of the setup for the FE test and later in situ preparation of the Pt shell. In the following experiments, the W probe would act as an anode and the MWCNT would play the role of a cathode, and both lead to the remote source meter. The gap between the tip of the W probe and MWCNT is defined as the working distance. During the FE process, the relative position of these electrodes can be modified with the movement of the two NRMs for working distance adjustment and tip alignment. 

**Figure 4:**
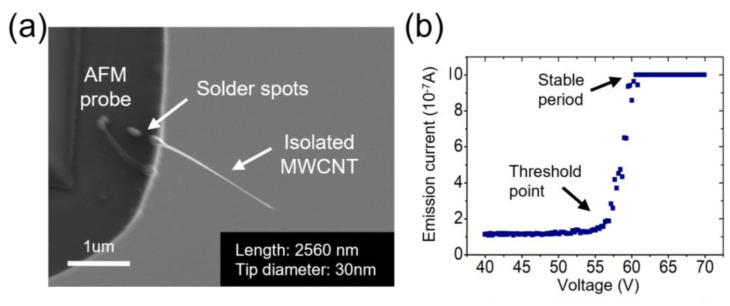
The initial status of the picked isolated MWCNT before the Pt shell growth, (**a**) an SEM image of the emitter; (**b**) the voltage versus current curve of the FE behavior of the isolated MWCNT.

Figure 4 shows the initial status of the chosen MWCNT for further preparation. This MWCNT has a tip with a radius of curvature of 20 nm and a length of 2.56 µm. Under a vacuum of $3\times 10^{-3}$ Pa, the working distance was set to 820 nm. A compliance current was set at 1 µA to avoid electrode burning caused by high currents. The bias applied on the electrodes swept from 0 V to 70 V with a step of 0.1 V. The voltage versus current is plotted in the inset of Figure 4, showing a typical FE characteristic. The current curve first appears as an approximately horizontal straight line on the order of 10 nA. When the voltage increases to 56 V, the current climbs rapidly and reaches the saturated value quickly. The current finally stabilizes at the stable current when the voltage increases to 62 V. This test was repeated many times to verify the reliability of the connection between the MWCNT and the AFM probe.

To prepare the hybrid nanowire, a FEID method was employed here, with a precursor of cyclopentadienyl-trimethyl-platinum (IV) (C_5_H_5_Pt(CH_3_)_3_) [32,33], which appears as a white crystalline powder at room temperature and has a vapor pressure of 45 mTorr [34]. The C_5_H_5_Pt(CH_3_)_3_ powder (STREM CHEMICALS, purity 99%, Newburyport, MA, USA) was filled into a pipette and placed one centimeter away from the MWCNT. When placed in an environment with low pressure, for example, the vacuum chamber of an SEM (in our experiments, the chamber vacuum was better than $4\times 10^{-3}$ Pa), the precursor will vaporize, providing an atmosphere with C_5_H_5_Pt(CH_3_)_3_. The C_5_H_5_Pt(CH_3_)_3_ molecules then diffuse and adsorb to the surface of the MWCNT. In the FEID process, the C–H bonds and Pt–CH_3_ bonds of the absorbed C_5_H_4_CH_3_Pt(CH_3_)_3_ molecules will be broken down for the emission-induced thermal effect on the tip of the isolated MWCNT [35,36], leading to the thermal decomposition of C_5_H_5_Pt(CH_3_)_3_ molecules [37]. The gas phase by-product including C and H elements will leave the surface of the MWCNT, finally forming a Pt shell [35]. The opening size of the pipette can change the density of the C_5_H_5_Pt(CH_3_)_3_ atmosphere and can be used to modify the growth rate of the Pt shell. For the FEID method, the precursor atmosphere is provided close to the electron source, enabling the formation of nanostructure on the cathode surface. However, in the FEBID method, as we used for welding the MWCNT to the AFM probe, the atmosphere is provided around the position under the irradiation of the electron beam, which is often far away from the electron source (such as the electron gun of an SEM), generating the deposition on the irradiated surface. So, the FEID method can be used for the in situ processing of the cathode, while the FEBID method is more focused on the phenomena that occur on the irradiated surface. 

**Figure 5:**
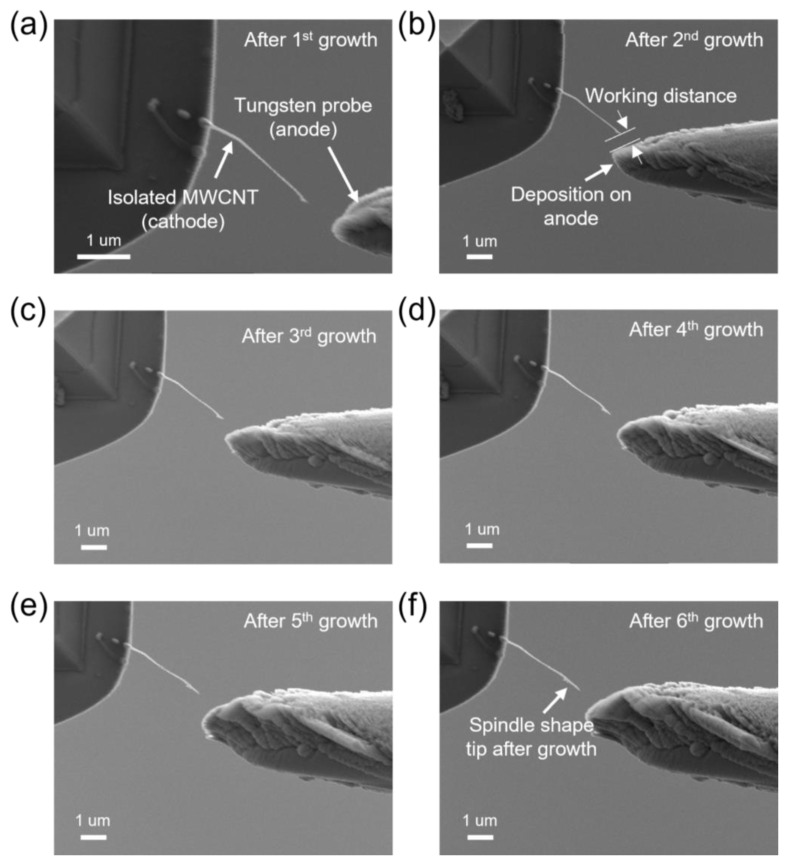
Growth of the Pt shell with the FEID method. (**a**) SEM image after the first two-minute growth, with the as-prepared isolated MWCNT acting as a cathode and the new tungsten probe acting as an anode; (**b**) SEM image after the second growth, the deposition can be found on the surface of the anode; (**c**–**e**) SEM images of the MWCNT after the third, fourth, and fifth growth; (**f**) image of the electrodes (the hybrid nanowire and the tungsten probe) after Pt shell preparation.

**Figure 6:**
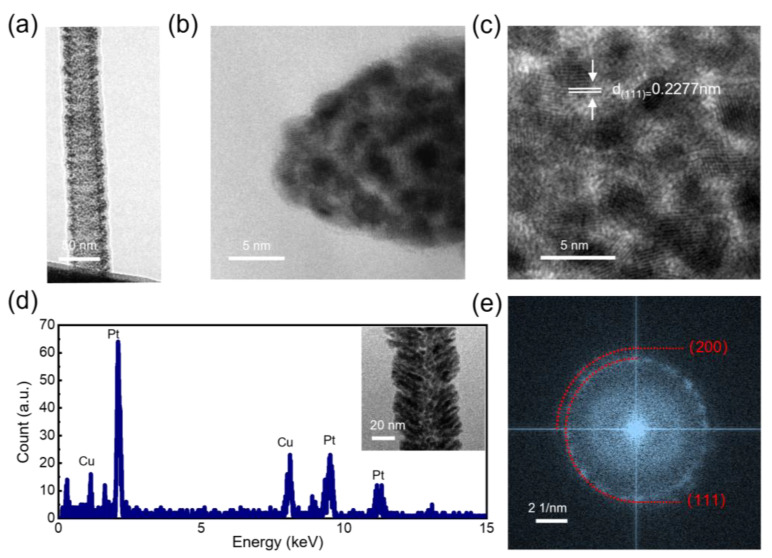
Characterization of the hybrid nanowire with the Pt shell. (**a**) TEM image on the side view of the nanowire, small Pt particles can be found on the surface of the MWCNT; (**b**) A TEM image of the tip of the as-prepared Pt shell; (**c**) high-resolution TEM image of the Pt particles on the tip with the characteristic lattice plane of (111) marked; (**d**) EDX spectrum of the Pt shell, the signals of copper (Cu) originating from the Cu grid for sample carrying; (**e**) FFT image of (**c**), with the polycrystalline rings of (111) and (200) marked.

At a working distance of 1 µm, we induced a 1 µA emission current from the MWCNT tip for the FEID-based growth of the Pt shell. The magnitude of the emission current can be achieved by adjusting the bias applied between the MWCNT and the W probe. This process was controlled by a specially designed program. A sweep voltage was first applied from 0 V and the emission current was precisely monitored. When the emission current reaches 1 µA (which means the field emission behavior happened), the voltage stops increasing. Once the current drops, the applied voltage will be raised automatically by the program until the emission current increases to 1 µA. The length of the Pt shell could be controlled by the time of growth. During the FEID, the electron beam of the SEM was blanked to avoid the effect of electron beam irradiation on imaging and both electrodes [38]. The growth process was divided into 2 min periods. Between each period, we quickly imaged the electrodes and adjusted their relative position to counteract the drift of the NRMs to maintain a stable working distance. After a total of 6 growth periods (12 min), an MWCNT-based hybrid electron source with a Pt shell was obtained. The experimental results of the growth process are shown in Figure 5. The grown Pt shell exhibits a spindle shape with a length of 750 nm and has a tip with a radius of curvature of 30 nm. The total length of the whole structure outside the AFM probe is about 3.3 µm after the preparation. A few depositions can be observed on the whole MWCNT, and the joint of the MWCNT and the AFM probe was also thickened, which can greatly contribute to the structural stability of the electron source during operation. Some deposition can be found on the surface of the tungsten, which coincided very well with the direction of the electrostatic field and is interpreted as an EBID phenomenon. Figure 6 shows TEM images and the characterization of the hybrid nanowire after preparation, with small Pt particles smaller than 5 nm distributed on the surface of the MWCNT. The TEM image in Figure 6b shows that the tip of the as-grown Pt shell is composed of loose polycrystalline Pt particles. Figure 6c shows a high-resolution TEM image of the Pt particles, with the characteristic lattice plane (111) marked. An energy Dispersive X-Ray Spectroscopy (EDX) analysis (Figure 6d) was carried out on the Pt shell, demonstrating the characteristic peak of Pt. Figure 6e shows a fast Fourier transform (FFT) image taken from Figure 6c; the polycrystalline rings of (111) and (200) prove the successful preparation of the Pt shell.

## 3. Results and Discussion

After the successful preparation of the Pt/MWCNT hybrid-nanowire-based electron source, fatigue tests were conducted inside an SEM. The basic setup is the same as in Figure 3, with the only difference being that the precursor for deposition was removed. The working current was set at 500 nA and also controlled by the program which was used in preparation. The upper working voltage was set to 200 V. 

**Figure 7:**
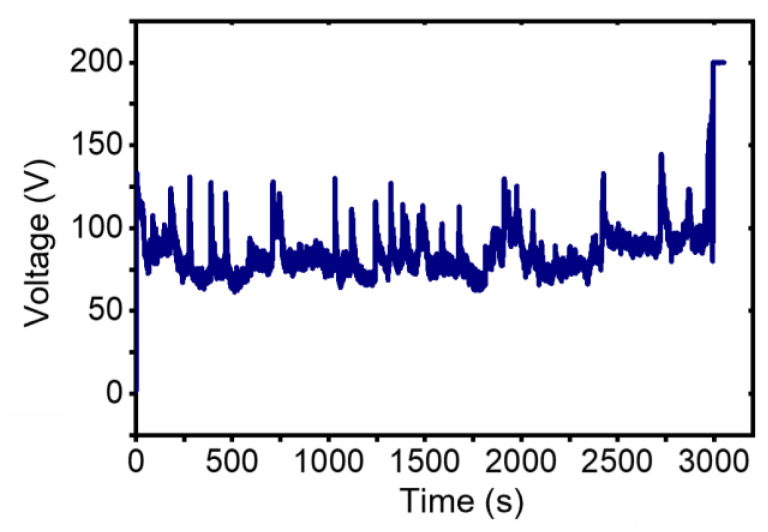
Working voltage versus time of a pristine MWCNT during a fatigue test.

A fatigue test was first conducted on a pristine MWCNT with a length of 3.2 um and a diameter of 35 nm. The working distance was set as 1 um and the chamber vacuum was better than $3\times 10^{-3}$ Pa. Figure 7 plots the graph of working voltage versus time (V-t) during the fatigue test. The MWCNT failed after working for 2994 s with a working current of 500nA. The fatigue test was carried out on different pristine MWCNTs many times and none of them has a lifetime of longer than 5 min.

A comparative test was then carried out on the as-prepared hybrid nanowire. During the fatigue test, the working vacuum was about $3.6\times 10^{-3}$ Pa at first and reached $2.26\times 10^{-4}$ Pa 25 h later. The whole test was divided into many periods for monitoring the situation of the electron source; each period lasted several hours. In this experiment, 34 periods made up the whole fatigue test. We set the working distance as 1um and corrected the distance between the electrodes after each period to achieve the set working distance of 1 µm. 

**Figure 8:**
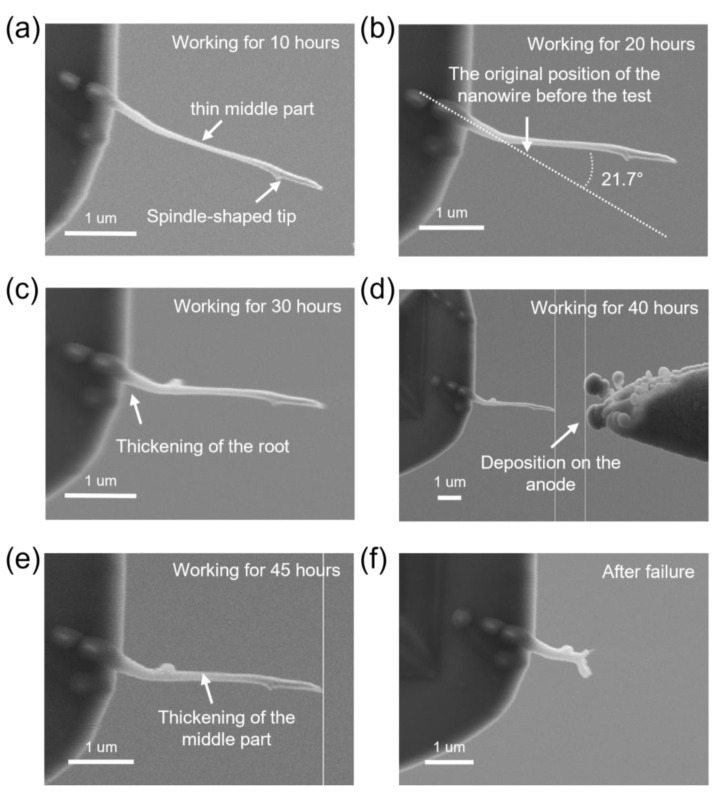
Morphological evolution of the hybrid-nanowire-based electron source during the fatigue test. (**a**) SEM image of the nanowire after working for 10 h, with a spindle-shaped tip and a thin middle part; (**b**) SEM image after working for 20 h—the nanowire is bent by 21.7°; (**c**) SEM image after working for 30 h—a thickening of the nanowire’s root can be found; (**d**) SEM image of electrodes after working for 40 h—some deposited structures appear on the surface of the anode; (**e**) SEM image of the nanowire after working for 45 h; (**f**) image of the nanowire after failure.

**Figure 9:**
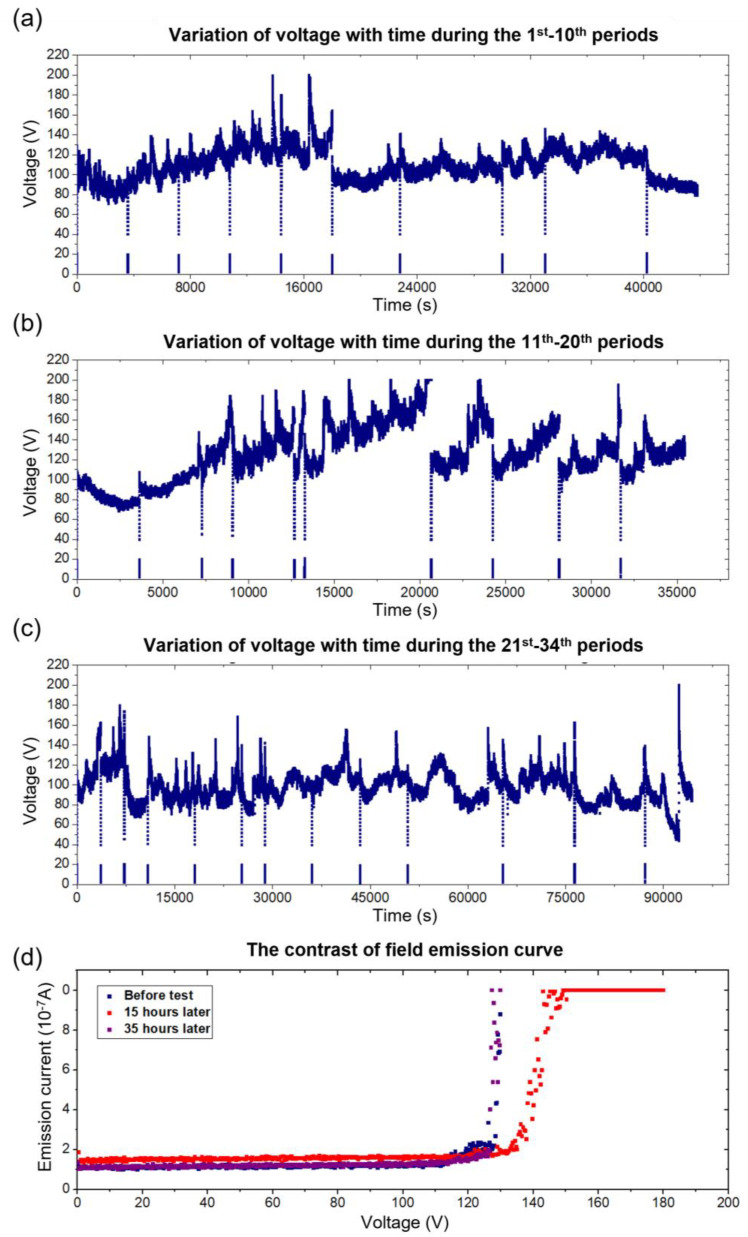
Variations in the working voltage and FE characteristics of our hybrid electron source during the test. (**a**–**c**) The time-dependent curve of working voltage during the 1st–10th periods, 11th–20th periods, and 21st–34th periods, respectively. (**d**) The contrast of the FE curve before, after 15 h, and after 35 h of the test.

Figure 8 depicts the morphological evolution of the electron source with an interval of 10 h during the fatigue test. A new W probe was replaced to act as an anode. In the beginning, the hybrid-nanowire-based electron source exhibits a relatively straight state, with an obvious shape of being thin in the middle and thick at the tip and the root. As the test progresses, the entire structure begins to thicken, and eventually, the thickness of the middle part approaches the thickness of the tip, as shown in Figure 8e. Some deposited structures are found on the anode surface, which is due to the FEBID phenomenon of ion clusters at low vacuum. Figure 8b shows the situation of electrodes worked for 20 h. A bending is observed on the electron source, and the direction of the hybrid nanowire is finally consistent with the direction of the electric field. This bending is thus attributed to the action of the electric field force. No noticeable influence of this bending was observed in the V–t curve in Figure 9d. Throughout the process, the structure of the electron source remained intact and mechanically stable. After more than 48 h of continuous work, the ion source finally burned out. 

The V–t curves of all periods are plotted in Figure 9a–c. After each period, the working voltage was turned off for imaging and working distance adjustment. Thus, the voltage-increase process was repeated many times, referring to the abnormal curve in Figure 9a–c. Throughout the test, the emission current stayed stable enough with the working voltage fluctuating around 120 V. The fluctuation is considered due to the changes in the working distance [39] from the jitter of the NRM under the electrical field. Additionally, it is important to consider the distance fluctuation brought on by “ballistic” emission [20]. A more stable electrode fixation method is needed for further research. 

The FE characteristic was investigated before the test, after 15 h, and after 35 h of the test and plotted in Figure 9d. The closeness of the threshold voltages at the three timepoints illustrates the constancy of the field emission characteristics during the fatigue test. After working for 24 h, the length of the hybrid nanowire outside the AFM cantilever is about 3.27 µm and the tip diameter increased to 47 nm. A total of 32 nm of the nanowire’s length was evaporated during the FE process, with an evaporation rate of 1.34 nm per hour. It is worth noting that the vacuum required for working is three orders of magnitude lower than that for published CNT-based emitters [1]. With five times the working current, the lifetime of the electron source has been increased by 100 times. Under the same working conditions (same setup and emission current), compared with the pristine MWCNT, its lifetime has been increased by more than 500 times. If this hybrid nanowire can work in a better environment (higher vacuum, fewer molecules), its lifetime can be further extended. 

As an important index of field emission devices, work function plays an important role in evaluating the device’s performance and studying material properties [40,41]. According to the Fowler–Nordheim theory, the work function of our hybrid nanowire was calculated by the $\ln\left(\frac{1}{V^{2}}\right)-\frac{1}{V}$ curve and the geometric enhancement factor β. The plot slope can be obtained from

$$K=\frac{\mathrm{d}\ln\left(\frac{J}{E^{2}}\right)}{\mathrm{d}\left(\frac{1}{E}\right)}=-6.83\times 10^{7}\Phi^{\frac{3}{2}}s\left(y_{0}\right),$$

where *J* is the density of the emission current, *E* is the electric-field intensity, Φ is the work function in eV, and $s\left(y_{0}\right)$ is a varying function derived from an elliptic integral and can be taken as constants varying between 1.00 and 0.833 [42]. It was stipulated that $s(y_{0})=0.917$ as the first-order approximation in our experiment. Our experimental results showed that the work function of the electron source increased obviously after the growth operation. The final value (5.34) was found between the value of the Pt (5.65) and a bare MWCNT (4.68), which indicated an enhancement phenomenon by modifying the energy state of the MWCNT. Tight bonds [43] between C atoms and Pt atoms make it easier for electrons to jump from the surface of the electron source, resulting in this enhancement. Furthermore, the Pt atoms on the surface of the electron source are thought to provide additional emission sites and contribute to the emission current simultaneously.

From the perspective of long-term working stability, our device has very obvious advantages in structural stability and low vacuum tolerance. Several reasons may contribute to its distinguishing properties. The adsorption of O_2_ molecules is the most common reason for device damage. The adsorption energy of O_2_ molecules on the Pt–Pt structure is 1.46 eV~2.16 eV [44,45]. However, the adsorption energy of O_2_ on CNT is only 0.68 eV [46]. Thus, during the emission process, the O_2_ molecules are more likely to adsorb to the Pt shell, reducing the degradation effect of gas-atom adsorption. This is likely to play a key role in the long-term fatigue test of our hybrid nanowire. In addition, large kinetic energy will be transferred to the C–C bonds on the CNT surface when electrons are emitted with a ballistic behavior [19], which refers to the tunneling behavior of electrons inside the CNT during emission, leading to a large evaporation rate and the rapid failure of CNTs during FE. However, due to the use of the NRMs, we control the working distance within 1um, which allows us to obtain a satisfactory emission current only by applying a small working voltage. Benefiting from the adsorption of Pt atoms, the structural stability of our MWCNT-based electron source is much improved, resulting in a better tolerance for the kinetic energy transferred from the emitted electrons.

## 4. Conclusions

In summary, an isolated long-time stable Pt/MWCNT hybrid-nanowire-based electron source was prepared with the FEID method. A 740 nm Pt shell was grown in situ at the tip of an MWCNT by using the NRMs inside an SEM. Benefiting from the protection of the Pt shell, the nanowire electron source showed excellent stability. A constant 500 nA current was achieved at low voltage (~120 V) under a poor vacuum ($3.6\times 10^{-3}$ Pa). The fatigue test indicates that the lifetime of our hybrid-nanowire-based electron source extended to more than 48 h, more than 500 times that of the pristine MWCNT emitter. The work function change before and after the Pt shell growth indicates the band-gap modulation of the MWCNT by the deposited Pt atoms. The Pt particles’ decoration also reduces the risk of O_2_ adsorption on the MWCNT’s surface and strengthens its structure, which is considered to be the reason for our hybrid-nanowire-based electron source’s capacity to withstand the poor working vacuum while showing excellent long-term stability. Our study simplifies the working requirement and dramatically improves the reliability of the MWCNT-based cathode. This method is predicted to lay a foundation for the fabrication of high-performance FE-based devices.

## Data Availability

Publicly available datasets were analyzed in this study.
